# Bibliographical

**Published:** 1894-12

**Authors:** 


### Bibliographical.

A Compend of Dental Prothesis and Metallurgy, by George W. Warren, D.D.S. with one hundred and twenty-nine illustrations.

This little work just issued from the press of P. Blakiston, Son & Co., has been prepared with special reference to the need of the dental student; and well does it fill the intention of its preparation. It presents the fundamental principles of dental mechanics, though brief, yet very direct, concise, and sufficiently full for the elementary work of the student. It covers the entire ground of prosthetic dentistry, and its study will enable the student to take up his initiative work with a good degree of efficiency, and well prepare him for the profitable study of more extensive works upon the subject. Such for example as: Richardson's Mechanical Dentistry, Evans' Crown and Bridgework, and the various works on Irregularities and their Correction.

In the cursory examination that we have made of the work, we will not venture a change in any particular, if the work is to be kept within its present compass. We can without qualification recommend it to the dental student and to the busy practitioner as well; to each it will serve as a book of reference that neither can well afford to be without.

